# Art Design Method of Industrial Products Based on Internet of Things Technology and Virtual VR

**DOI:** 10.1155/2022/9666827

**Published:** 2022-06-14

**Authors:** Yu Yu

**Affiliations:** School of Design and Creativity, Fujian Jiangxia University, Fuzhou, Fujian 350108, China

## Abstract

The Internet of Things technology transmits and shares physical information on the Internet, and describes the continuous world through scattered data. From the perspective of internal characteristics, the efficient interconnection of large-scale heterogeneous network elements is composed of the Internet of Things and imported from abroad, so there has been a high degree of accumulation in foreign countries so far. In 1980, the United States proposed a virtual reality concept called VR for short. Embedded virtual VR technology usually uses equipment to close the user's senses and temporarily isolate the user from the actual environment. This article also discusses the design of industrial products, the continuous development of big data, artificial intelligence, and additive material manufacturing technology to make the design methods and manufacturing processes of industrial products more scientific and systematic, and promote the diversity and precision of industrial product design. Social informatization plays an important role in all areas of digital technology design. Parametric design has changed the design thinking mode of designers and formed new design trends and styles. Taking the search for relationships and rules as the starting point of the design and the algorithm as the core content of the design are very different from traditional design ideas. The computer age has not only cultivated new styles but also cultivated new artistic design techniques for industrial products. Designers should strive to explore the potential functions of algorithm technology and focus on smarter and more logical design and production processes. In this paper, through the research of the Internet of Things technology and virtual VR, it is applied to the research of the artistic design method of industrial products, aiming to promote its better development.

## 1. Introduction

Beginning in 1999, the Massachusetts Institute of Technology in the United States began to study the Internet of Things in the laboratory. In 2005, the International Telecommunication Union officially defined the concept of the Internet of Things. The goal of information and communication technology has evolved from people-to-people exchanges to connections and mutual exchanges between people and things, and between things and things [1]. The Internet era of Internet of Things technology with all things interconnected is about to begin [2]. In addition, we also studied virtual reality technology. Virtual reality technology is a computer simulation system that can create and experience a virtual world [3]. By using a computer to generate a simulation environment, users can have a sense of immersion in the environment [4]. Virtual VR technology combines virtual VR technology with various output devices through the use of data used in real life and electronic signals generated by computer technology to transform it into a phenomenon that people can feel [5, 6]. These phenomena are objects in reality, or matter that we cannot see, and they are displayed in front of us by three-dimensional models [7]. Because we cannot directly see these phenomena through the naked eye, but need to simulate the real world by computer technology, it is also called virtual reality [8]. This article also discusses the work on industrial products and their design. At the beginning of the industrial revolution, the esthetic issues of industrial products attracted the attention of many scholars and companies. Social practice has proved that esthetic value is an indispensable part of successful products [9]. After the twenty-first century, esthetic culture has become more and more prevalent, especially the development of culture and creative industries, which comprehensively promotes the continuous development of the state of esthetic values [10]. Today, under the guidance of the national innovation strategy, various industries are innovating product design and supply-side reform through more digital, networked, and intelligent technologies [11]. Under the new circumstances, the esthetic value of the product has undergone major changes in both its connotation and extension [12]. The esthetic value of a product is not only an incidental value but also a spokesperson for product quality. With the continuous development of science and technology and social economy, social material production has become more and more abundant [13]. The concept of product design and production has shifted from centering on “manufacturing as the direction, product as the center” to “market as the direction and user as the center” trend. The consumer's concept of consumption has shifted from focusing on basic performance to focusing on esthetic characteristics and experience [14]. Most consumers evaluate and choose most products from an esthetic point of view, and consumers' esthetics and judgment have been double-improved [15]. In this context, the combination of the innovation of art design and the esthetic economy has become the most important productive force for social development [16].

## 2. Related Work

The literature introduces the origin and development of the Internet of Things, introduces the inevitability of the meaning of the Internet of Things, and then focuses on the hierarchical service model of the Internet of Things ontology, and introduces in detail the architecture of the intermediate links of the Internet of Things based on the ontology [17]. The literature puts forward the problems of IoT service discovery, analyzes the service discovery process based on its meaning, and discusses the semantic orientation, classification, and matching calculation methods of IoT service discovery [19]. The literature shows that taking the wiring shell as an example, the process structure of the part is analyzed. Based on the mold flow analysis, the best molding process parameters of the part are determined through experiments, combined with the structural characteristics and parameter analysis, and calculation and inspection are used for the molding of plastic parts [20]. The key components of the inlaid side core injection model have been designed and gradually calculated and corrected for its pouring system, molded part mechanism and cooling mechanism, side core mechanism, ejection system, and other related structures, completing the finite element correction of cavity load finally; the working principle of the mold and the simulation of the mold opening and closing movement process were completed; and the overall design of the wiring shell mold structure was realized [21, 22]. The literature introduces the architecture and mode of the intermediate links of the Internet of Things, and discusses the reasoning based on the formalization of rules and ontology knowledge, and finally proposes a dynamic service mode based on the context of the ontology [23]. The literature shows that for molds with complex structures and high assembly accuracy requirements, the method of establishing physical prototypes is used in design assembly engineering. The design requires repeated assembly and changes, and the modification process needs to rely on the physical model. Through virtual assembly technology, a VR intelligent system for mold assembly that combines VR equipment and data gloves is developed. A virtual prototype of a complex structure and high-precision design mold is constructed in a virtual space, and it is applied to physical drag-and-drop assembly [24]. The designer himself can perform perceptual identification verification on the rationality and standardization of the product design, the possibility and difficulty of assembly, and other factors [25].

## 3. Internet of Things and Virtual VR Technology

### 3.1. Internet of Things

**Figure 1:**
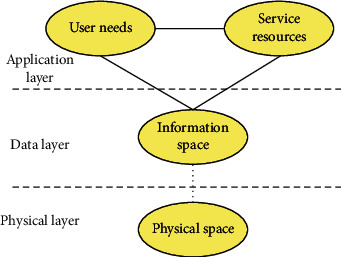
Information interaction model of the internet of things.

In this article, we have studied and analyzed the significance of the IoT architecture model, and proposed a semantic-based IoT service system model to realize the regional positioning of the IoT. Considering the diversity of IoT devices, this model integrates the IoT divided into physical space, information space, user needs, and service resources; IoT service resources are reflected in the actual physical space through the operation of the information space; and changes in the physical space are transmitted to the information space, affecting users and IoT services. The interaction model of user needs, service resources, physical space, and information space on the Internet of Things is shown in Figure 1.

### 3.2. Virtual VR Technology

Sphere form: the sphere is the most effective and flexible 3D model. It is defined as the smallest volume sphere that can wrap an object. On the surface of the center and radius of the sphere, we measure the distance between all the end points of the part to find the maximum value, that is, the radius of the sphere. The half point of this line is the center of the sphere.

(1) $$\begin{array}{c} \text{R}=\left\{\left(\text{x},\text{y},\text{z}\right)|{\left(\text{x}-{\text{O}}_{\text{x}}\right)}^{2}+{\left(\text{y}-{\text{O}}_{\text{y}}\right)}^{2}+{\left(\text{z}-{\text{O}}_{\text{z}}\right)}^{2}<{\text{r}}^{2}\right\}. \end{array}$$

The formula of ball center is as follows:

(2) $$\begin{array}{c} {\text{O}}_{\text{x}}=\frac{1}{2}\left({\text{x}}_{\text{max}}+{\text{x}}_{\text{min}}\right),{\text{O}}_{\text{y}}=\frac{1}{2}\left({\text{y}}_{\text{max}}+{\text{y}}_{\text{min}}\right),{\text{O}}_{\text{z}}=\frac{1}{2}\left({\text{z}}_{\text{max}}+{\text{z}}_{\text{min}}\right). \end{array}$$

The formula of ball radius is as follows:

(3) $$\begin{array}{c} \text{r}=\frac{1}{2}\sqrt{{\left({\text{x}}_{\text{max}}-{\text{x}}_{\text{min}}\right)}^{2}+{\left({\text{y}}_{\text{max}}-{\text{y}}_{\text{min}}\right)}^{2}+{\left({\text{z}}_{\text{max}}-{\text{z}}_{\text{min}}\right)}^{2}}. \end{array}$$

The detection of the intersection of the spheres is relatively simple, and only the relationship between the center distance and the radii of the two outer spheres needs to be determined. If the formula is valid, the overlap of the balls will cause collision detection.

(4) $$\begin{array}{c} \left|{\text{c}}_{1}{\text{c}}_{2}\right|<\left({\text{r}}_{1}+{\text{r}}_{2}\right). \end{array}$$

The AABB bounding box along the coordinate axis is the most commonly used detection method in the research of collision detection algorithms. Since the AABB bounding box is composed of the smallest hexahedron whose edges are parallel to the coordinate axis, only six scalars are needed to construct the AABB bounding box. The formula for the radius of the center position is as follows:

(5) $$\begin{array}{c} \text{R}=\left\{\left(\text{x},\text{y},\text{z}\right)\|{\text{x}}_{\text{c}}-\text{x}\left|\leq {\text{r}}_{\text{x}},\right|{\text{y}}_{\text{c}}-\text{y}\left|\leq {\text{r}}_{\text{y}},\right|{\text{z}}_{\text{c}}-\text{z}|\leq {\text{r}}_{\text{z}}\right\}. \end{array}$$

The formula of the ball center is as follows:

(6) $$\begin{array}{c} {\text{x}}_{\text{c}}=\frac{1}{2}\left({\text{x}}_{\text{max}}+{\text{x}}_{\text{min}}\right),{\text{y}}_{\text{c}}=\frac{1}{2}\left({\text{y}}_{\text{max}}+{\text{y}}_{\text{min}}\right),{\text{z}}_{\text{c}}=\frac{1}{2}\left({\text{z}}_{\text{max}}+{\text{z}}_{\text{min}}\right). \end{array}$$

The formula of ball radius is as follows:

(7) $$\begin{array}{c} {\text{r}}_{\text{x}}={\text{x}}_{\text{c}}+\frac{1}{2}{\text{x}}_{\text{min}},{\text{r}}_{\text{y}}={\text{y}}_{\text{c}}+\frac{1}{2}{\text{y}}_{\text{min}},{\text{r}}_{\text{z}}={\text{z}}_{\text{c}}+\frac{1}{2}{\text{z}}_{\text{min}}. \end{array}$$

The OBB bounding box is also called the direction bounding box. However, collision detection calculations are relatively complicated, and OBB has good agility. It can make real entities better to create and help to better complete collision detection. The formula for the direction bounding box area is as follows:

(8) $$\begin{array}{c} \text{R}=\left\{\text{O}+{\text{ar}}_{1}{\text{v}}_{1}+{\text{ar}}_{2}{\text{v}}_{2}+{\text{ar}}_{3}{\text{v}}_{3}|\text{a},\text{b},\text{c}\in \left(-1,1\right)\right\}. \end{array}$$

Here, O represents the center of the OBB, and *v*_1_, *v*_2_, and *v*_3_ can determine the vector target whose direction bounding box positively intersects with the absolute coordinates.

(9) $$\begin{array}{c} {\text{a}}_{\text{XY}}=\frac{1}{3\left(\text{n}-1\right)}\overset{\text{n}}{\underset{\text{i}=1}{\sum }}\left({\text{M}}_{x}^{i}-{\text{U}}_{\text{x}}\right)\left({\text{M}}_{Y}^{i}-{\text{U}}_{\text{Y}}\right),\\ {\text{C}}_{3\times 3}=\left({\text{a}}_{\text{mn}}\right)\text{m},\;\text{n}\in \left\{\text{X},\text{Y},\text{Z}\right\},\\ \text{U}=\frac{1}{3\text{n}}\overset{\text{n}}{\underset{\text{i}=1}{\sum }}\left({\text{M}}^{\text{i}}+{\text{N}}^{\text{i}}+{\text{K}}^{\text{i}}\right). \end{array}$$

Through actual verification, by increasing the cross-sectional area of the gating system, the warping deformation of the plastic parts can be reduced from 0.373 to 0.370, the effect is not obvious, and there is no need to increase the cross-sectional area of the gating system. Therefore, the three performance parameters that have a great influence on warpage as shown in Table 1 are designed as variables for the MJ1504 wiring housing for process optimization.

In this experiment, the optimization method shown in Table 2 is used as the conditional boundary of the wiring housing, the melting temperature, and the cooling material index are used as variables, and the cooling time and warpage deformation are used as the quality benchmark. The purpose is to reduce the cooling time and improve the production efficiency of the product by adjusting the target variable while ensuring the slight warpage of the plastic part.

**Figure 2:**
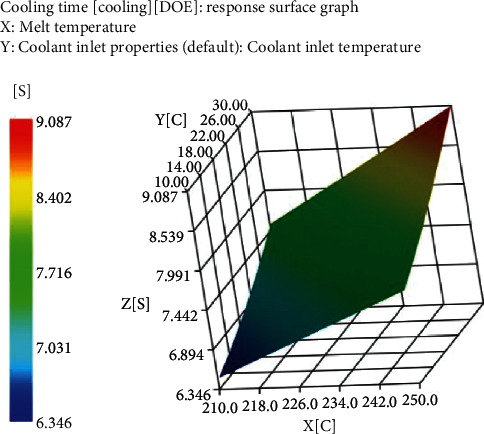
Cooling time response surface.

As shown in Figure 2, under certain cooling water temperature conditions, as the melting temperature increases, the cooling time of plastic parts increases significantly; at the same melting temperature, the cooling time can be greatly shortened by reducing the cooling water temperature. Therefore, in order to shorten the cooling time of plastic parts, it is mainly to reduce the molding temperature and the inlet temperature of the cooling material, and to strengthen the cooling.

**Figure 3:**
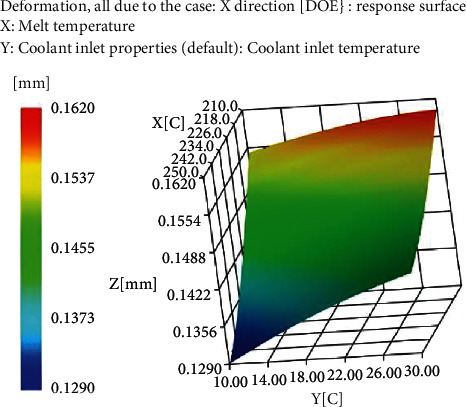
Warping deformation response surface in *X* direction.

By comparing the deformation of the plastic part in the *x*, *y*, and *z* directions, it is found that the deformation of the plastic part is mainly in the *x* direction. It is found from the response surface that the melting temperature has the greatest influence on the uneven shrinkage and deformation of plastic parts. On the premise that there is no change in other molding conditions, by increasing the melting temperature, uneven shrinkage and deformation can be reduced, and at the same time, by reducing the temperature of the cooling material, the deformation of the plastic part can be greatly reduced, as shown in Figure 3.

**Figure 4:**
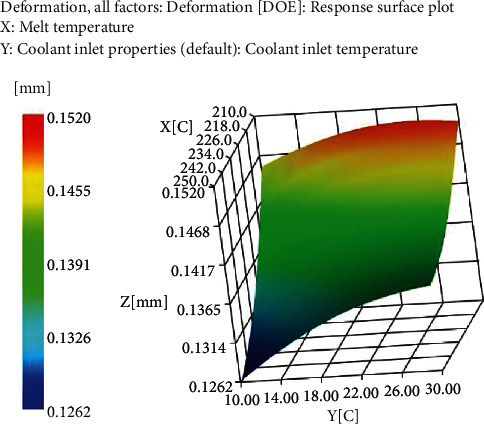
Warpage response surface of all factors.

The warpage deformation response surface is shown in Figure 4.

The casting mold is subjected to various pressures during casting. If the strength of the cavity is not enough, the plastic parts will be deformed or cracked. When the rigidity is insufficient, under the action of elastic deformation, the joint surface of the mold is deformed, and cracks caused by flash burrs. After the forming pressure disappears, the mold elastically recovers, causing serious shrinkage, which affects the quality of the plastic parts and destroys the plastic parts. Therefore, the mold design must meet the requirements of strength and rigidity.

We calculate the thickness of the mold cavity wall according to the formula:

(10) $$\begin{array}{c} {\text{S}}_{\text{c}}=\sqrt[{3}]{\frac{{\text{cpa}}^{4}}{\mathrm{E\delta}}}. \end{array}$$

We determine the thickness of the cavity bottom plate according to the formula:

(11) $$\begin{array}{c} {\text{S}}_{\text{h}}=\sqrt[{3}]{\frac{{\text{cpb}}^{4}}{\mathrm{E\delta}}}. \end{array}$$

The thickness of the dynamic mold support plate directly affects the strength and rigidity of the cavity bottom plate. It is necessary to calculate the force of the dynamic mold support plate and design its thickness. We calculate the thickness of the support plate according to the formula:

(12) $$\begin{array}{c} h=K\sqrt{\frac{FL}{2B\left[\sigma \right]}}=K\sqrt{\frac{ApL}{2B\left[\sigma \right]}}=0.75\times \sqrt{\frac{45\times 18557\times 294}{2\times 450\times 200}}=38\text{mm.}\\ \text{h}=\text{K}\sqrt{\frac{\text{FL}}{2\text{B}\left[\sigma \right]}}=\text{K}\sqrt{\frac{\text{ApL}}{2\text{B}\left[\sigma \right]}}. \end{array}$$

We calculate the tensile force of the core-pulling mechanism according to the formula:

(13) $$\begin{array}{c} \text{Q}={\text{lnp}}_{2}\left({\text{f}}_{2}\mathrm{cos\theta}-\text{sin}\theta \right). \end{array}$$

For safety reasons, the pulling-out distance of the side core is usually greater than the depth of the undercut structure, so that the core-pulling mechanism can completely pull out the undercut structure. We calculate the core-pulling distance according to the formula:

(14) $$\begin{array}{c} \text{S}=\text{H}+\left(2∼3\right). \end{array}$$

In the core-pulling process, the tensile force and the inclination angle of the inclined guide column directly affect the bending force. The bending force is calculated according to the formula as follows:

(15) $$\begin{array}{c} \text{P}=\frac{\text{Q}}{\cos\alpha }. \end{array}$$

We calculate the diameter of the inclined guide column according to the formula:

(16) $$\begin{array}{c} \text{d}=\sqrt[{3}]{\frac{\text{PH}}{0.1\text{cos}\alpha \left[\sigma \right]}}. \end{array}$$

We calculate the total length of the inclined guide column according to the formula:

(17) $$\begin{array}{c} \text{L}=\frac{\text{h}}{\cos\;\alpha^{\circ }}+\frac{\text{S}}{\sin\;\alpha^{\circ }}+\frac{1}{2}{\text{Dtan}\alpha }^{\circ }+\left(5∼10\right). \end{array}$$

The melting temperature of plastic casting is about 230°C, and the temperature of the plastic part that reaches the peak condition is about 88°C. A lot of heat is released during the molding process. In order to reduce the temperature of plastic parts and molds, the natural cooling effect is poor and the time is long, so in order to speed up the cooling rate, a cooling system needs to be set.

We calculate the volume flow of heat and cooling water according to the formula:

(18) $$\begin{array}{c} {\text{q}}_{\text{v}}=\frac{{\text{WQ}}_{1}}{{\rho c}_{1}\left(\theta_{1}-\theta_{2}\right)},\\ {\text{Q}}_{1}={\text{c}}_{2}\left(\theta_{3}-\theta_{4}\right)+\text{h.} \end{array}$$

The terminal housing is a thin-walled section with a rectangular cross section. We calculate the lock tension according to the formula:

(19) $$\begin{array}{c} \text{F}=\frac{8\delta_{2}\text{ESlcos}\varphi \left(\text{f}-\text{tan}\varphi \right)}{\left(1-\mu \right){\text{k}}_{2}}+0.1\text{A.} \end{array}$$

We calculate the diameter of the push rod according to the formula:

(20) $$\begin{array}{c} \text{d}=\psi {\left(\frac{{\text{L}}^{2}\text{F}}{\text{nE}}\right)}^{1/4}. \end{array}$$

We check the strength of the putter. The material of the push rod is T8a, and the allowable stress is 120 MPa. We use 20 push rods on a plastic part according to the formula:

(21) $$\begin{array}{c} \sigma_{\text{c}}=\frac{4\text{F}}{{\text{n}\pi \text{d}}^{2}}\leq \left[\sigma \right]. \end{array}$$

The marked dimensions and dimensional tolerances of plastic parts are shown in Table 3.

The radial dimensions of the cavity and core are shown in Table 4.

The maximum and minimum displacement nodes of the cavity in the *x*, *y*, and *z* directions are represented by cloud diagrams. As shown in Table 5, the maximum displacement of the cavity node is 0.007 mm, and the deformation is very small.

## 4. Industrial Product Art Design Method and Application Research

### 4.1. Parametric Design of Industrial Products

**Figure 5:**
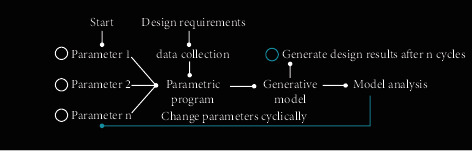
Flowchart of parametric design.

Parametric is a term widely used in various fields from mathematics to design. Specifically, it refers to the calculation of a predetermined value. Parametric design mainly refers to the way of design thinking, rather than using some software or some modeling techniques. It is based on mechanical design, and industrial designers draw on its thinking mode. The conventional design method is implemented in stages. If the plan is changed, it needs to be redesigned from scratch. Parametric design reveals many factors that affect the design form, describes the inherent logical relationship of computer language, and constructs a parametric model on a digital platform. In parametric design, parameters reflect the logical relationship between design elements. Changes to any one component will affect its related components. The relationship between components is dynamic and changes with the change of design conditions, as shown in Figure 5.

With the advancement of digital technology, it provides a source of power for design conversion, and the design software is always upgraded with the advancement of hardware technology. Software developers include many functional functions and logical rules. Designers can quickly create logical relationships between variables as needed and generate necessary design results by controlling predesigned rules. The use of parametric design software leads to the uncertainty and complexity of the design, and simulates the complex natural phenomena in the evolution of logic. Based on the visual programming platform of industrial aided design, the complete model generation logic is constructed through a series of modular calculation units, which are usually used for the visual performance of animation. The main modeling method is polygon modeling, when it is used for parameter design at the same time, it must use the basic programming language. A certain mathematical calculation software is usually used for complex mathematics and engineering problems. The programming language used for parameter design has strong expansion capabilities, but it is difficult for general designers to master it.

### 4.2. Parametric Design Elements

**Figure 6:**
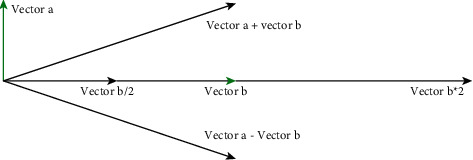
Illustration of basic vector operations.

Points and vectors are the basic elements of three-dimensional space and the basis of parameter design. In geometry, a point represents a position in space. A vector is an abstract element with a direction and length, and has no fixed position. Vectors and points are represented by (*x*, *y*, *z*), but they are different. Vector calculations are often used in parameter design, as shown in Figure 6.

There are three ways to form the surface of the polygonal mesh through the parametric design method. In the first type, the mesh surface is generated by topology, and the vertices determine the topology of the mesh surface. A mesh surface can be generated from multiple points by components, but manual point distribution is very troublesome. Usually, the vertices and components of the existing model are combined and extracted to generate a new mesh surface. The second is to generate a mesh surface through the triangular surface algorithm. This algorithm can maximize the internal angle of the generated triangles, thereby uniformly generating a reasonable triangle mesh surface to prevent the surface of the triangle from becoming smaller.

Most of the research on curves and surfaces is about how to generate curves and surfaces. In the parameter control generation method, it is very important to study the analysis method of curve and surface. Parameter design can repeatedly adjust the shape of the curve according to the operating curve data and continuously optimize the function parameters and shape parameters to meet the design requirements. This is a new curve design method. The Nurbs curve is the basis for the construction of the shape and is a derivative of the previous Bezier curve. The algorithm of the Nurbs curve is more complicated than the Bezier curve, but the control is also easier. Only one parameter *T* of the curve is one-dimensional, and the two-dimensional surface has two parameters *U* and *V*, respectively. The curve is divided into positive and negative directions, and its surface is equivalent to the vertical direction *W*. Compared with the curvature of the curve, the curvature of the curved surface is not intuitive. The curvature of the point *P* on the curved surface refers to the deviation from the tangent plane of the point *P*. We use the point *P* to make several sections and calculate the point *P* at the intersection of the section and the surface. Because countless sections passing through point *P* can be made, there are countless curvatures *K*, the highest curvature is *K*1, and the lowest curvature is *K*2. Gaussian curvature and minimum curvature are two important surface theories. The Gaussian curvature formula is *g* = *k*1 ∗ *k*2. When the Gaussian curvature is greater than 0, the surface is convex, and when the Gaussian curvature is less than 0, the surface is concave.

### 4.3. Data Structure and Processing

Data collection is the preliminary work of parameter design. Through investigation, all design requirements are digitized to form parametric variables. The starting point of the design is to meet the design requirements of human actions. The data of this information can change the design requirements into parameters that can be recognized by the computer, which is the basis for parameter generation. Data structure is the core of parameter design and the form of parameter storage. The deeper the design logic, the more complex the data structure. Different platforms have different data structure processing methods. In parametric modeling software, tree data is the core concept of platform data operation. This data structure includes multiple parallel data list groups, which can separately process any data in the group. Linear data is the simplest data structure; that is, sequence groups are sorted and placed in lists and then divided into long lists, short lists, and crossover operations. The long list is the default algorithm of Grasshopper. Tree data is similar to nested lists and consists of multiple parallel data lists. The arithmetic processing of tree data is actually to process linear data list operations of multiple lists in order. The two core algorithms of the overall data structure are the long list algorithm and the sequence correspondence operation. Two ordinary data must be correspondingly operated one by one. If the tree data corresponds to the normal data, all the normal data is used to calculate each group of the tree data, and the tree data is calculated correspondingly within the group.

### 4.4. Art Design Path of Industrial Products

Positioning refers to the company's unique value positioning in the minds of target customers by designing its own product image. Positioning is not to focus on the target of the product object but what the target consumer wants to do. By speculating on the consumer's psychological needs, how to emphasize the consumer's product differentiation in a limited psychological space is the criterion for judgment. The esthetic value of the product is an important overall plan and strategy formulated by the company to pursue and maintain a lasting competitive advantage as the basic point of the company's future. The overall strategy is divided into three levels: overall, business units, and functions. According to the different levels of corporate strategy and the state of corporate design innovation, the design strategy can be divided into three levels: overall design, competitive design, and functional design. What kind of design innovation strategy an enterprise adopts and how to execute the strategy must be determined by analyzing the opportunities and threats of the external environment, and the advantages and disadvantages of internal capabilities according to the company's development goals.

The development of an enterprise is inseparable from strategic guidance. Different environments and their own conditions determine that companies need to adopt different strategies to maintain long-term development. As a design strategy, the design innovation of product esthetic value requires different choices under different circumstances.

A stable strategy means that the company maintains the same development model and development goals as in the past, and the commercial scope of products and services is basically unchanged. When industrial technological innovation is at a mature stage, when consumer preference goals and intra-industry competition tend to stabilize, companies generally adopt this strategy. Under the stable strategy, the focus of design innovation is to maintain the combination and design direction of the original product design, and change the design style and form. The esthetic value of a product is to maintain the original brand image of the product, and to meet the basic esthetic needs of consumers is to change the design form.

Expansion strategy means that companies continue to increase their business and scope and increase their market share. Development is the instinct for survival, and most companies will go through this strategic period. Under the guidance of this strategy, companies generally achieve expansion goals through horizontal integration, vertical integration, and various developments. Under the expansion strategy, the design innovation of the company is very active, the product design is diversified, and the design organization is flexible and diverse. Designing a good product is favored by more markets and consumers. Understanding the corporate brand image is the core of corporate design innovation in this period. Therefore, the esthetic value of products plays an important role in design innovation. At this point, product esthetic value design innovation activities should be very active. It requires breakthrough innovations in different consumer target markets from multiple levels, and esthetic value design innovation from multiple levels.

The shrinking strategy means that the company retreats or shrinks from the existing business area, concentrates resources to respond to environmental changes, and maintains the company's survival. The shrinkage strategy is a negative strategy, generally a short-term strategy adopted during the period when the external environment is strongly impacted or when the enterprise is facing a downturn. The contraction strategy can be divided into active contraction and passive contraction. Under the active contraction strategy, product esthetic value design innovation must find the right position, emphasize the characteristics of the product, and increase the value of the product to consumers. Under the passive strategy, product esthetic value design innovation needs to reduce the enterprise's resource investment and maintain product image by integrating micro- and external resources. Due to the functionality of the main product, in some cases, companies can abandon the design innovation of the esthetic value of the product.

The competitive analysis framework crosses the boundaries of industries, specific technologies, or management methods. The emergence of the Internet has changed the barriers to industrial participation, redefined the power of buyers, and promoted the emergence of new alternative models. On the other hand, the basic driving force of industrial competition remains unchanged.

The cost leadership strategy means that the company reduces operating costs and product costs as much as possible, and takes low prices, small profits, and high profits as its overall goals. In order to achieve the total cost, it is necessary to comprehensively manage the human, material, and financial resources of the enterprise, and formulate a series of control and optimization plans in the process of product research, trial production, production, and sales. Reduce experience costs and indirect costs, and achieve efficient production. Total cost leadership is a cost management strategy, and the ultimate goal is to achieve the largest sales of products on the market. In order to curb costs, if product quality is reduced, the importance of cost leadership will be lost. The design innovation of product esthetic value can not only directly reduce the cost of products but also indirectly reduce the relative cost of products. In the context of the declining natural resources of the era, it provides us with a powerful set of tools to survive and develop. Minimalist style is the world's mainstream esthetic style. It is not only an economic method to achieve low prices and high value, but also a method to promote environmental protection by reducing materials and unnecessary functions. The minimalist style reduces manufacturing costs such as the complexity of manufacturing raw materials, production procedures, and production processes by modeling simplified design, modulus design, and standardized design of constituent elements. Although the total product cost will rise with the design innovation, the esthetic value of the product increases the overall value of the product, so the design innovation of the product esthetic value is valuable. Through the strategy of leading total cost, a domestic mobile phone brand continues to innovate product designs, quickly occupy the market, and quickly grow into one of China's top mobile Internet technology companies to achieve high cost performance.

Differentiation strategy refers to the difference between an enterprise and a competing company in terms of brand positioning, product style, and operation mode. Due to the development of the times, the differentiation strategy of enterprises has experienced three development stages: product differentiation, image differentiation, and operation mode differentiation. Samsung Electronics is the largest electronics industry in South Korea. In terms of differentiation strategy, it mainly conducts product differentiation management through independent research and development, including breaking three-dimensional thinking, vertical integration, and strong cooperation. Not the same, improving the supply chain and circulation. Generally speaking, companies can use four methods to reflect product differentiation. First, high quality means that the product is more durable, safer, and more stable than other companies. A well-known mobile phone brand has excellent quality, stable signal, and good quality characteristics; the second is beautiful design, which is more reasonable and more beautiful than other companies' products. A well-known brand attaches great importance to product design, emphasizing the grade, and quality of the product; third, higher cost performance, that is, the ratio of the product function perceived by consumers to the quality of the purchase price is very high. The low price strategy is a common method to achieve high cost performance, but the high performance of the product does not necessarily depend on low prices. Fourth, new concepts, that is, products bring new cultural experiences and new lifestyles. This approach often brings breakthrough innovation, which is the combined effect of technology, design, and culture. The differentiation of the design innovation of the esthetic value of the product means that the difference between the products of the enterprise and the competing companies needs to be realized through one or more methods such as difference in perception, difference in function experience, or difference in image. Through the differentiated design of products, companies can not only avoid direct competition with competitors but also enrich product categories and provide consumers with more choices.

## 5. Conclusion

At present, molds with complex structures and assembly precision need to adopt the method of establishing physical prototypes to design the assembly process. Therefore, it is necessary to repeatedly assemble and change the design, and rely on the physical model in the change process. Through virtual assembly technology, a mold assembly virtual VR system that combines VR equipment and data gloves is developed. A virtual prototype of a complex structure and high-precision design mold is constructed in a virtual space, and it is applied to physical drag-and-drop assembly to allow design. The author can conduct verifiable research on the rationality and standardization of product design, the possibility and difficulty of assembly, and other factors. The esthetic value of a product is based on a specific historical and cultural background, taking material as a carrier, through design innovation or cultural accumulation, so that the artistic design of the product can meet people's esthetic needs, and bring people the intangible value of esthetic pleasure and spiritual enjoyment. The esthetic value of the product has the basic characteristics of esthetic entertainment, value dependence, rapid observation, diverse forms, and dynamic changes. Material factors, technical factors, and spiritual factors are the three major bearing factors of product esthetic value, from the macro social environment to the research and development of related enterprises, production, and sales, and microconsumption behavior will affect the esthetic value of the product.

## Figures and Tables

**Table 1:** Process plan comparison.

Performance parameter	Original plan	Scheme 1	Scheme 2	Scheme 3
Melting temperature (°C)	240	240	240	210
Mold temperature (°C)	90	90	90	90
Injection time (s)	4	4	4	4
Injection + holding pressure + cooling time (s)	48	48	48	42
Maximum holding pressure (MPa)	80	100	100	80
Holding time (s)	20	20	25	20
Warpage (mm)	0.373	0.209	0.199	0.405
Sink mark (mm)	0.186	0.077	0.078	0.176
Scheme variables	—	Increase the holding pressure	Pressurize, extend the holding time	Reduce melt and mold temperature

**Table 2:** DOE optimization process plan.

—	Scheme 1	DOE optimization 1	DOE optimization 2
Melt temperature (°C)	240	210	210
Mold temperature (°C)	90	60	60
Coolant inlet temperature (°C)	30	30	20
Maximum holding pressure (MPa)	100	80	100
Holding time (s)	25	20	20
Injection + holding pressure + cooling time (s)	48	39	31
Deformation (mm)	0.209	0.405	0.231
Sink mark (mm)	0.077	0.176	0.129
Scheme variables	—	Melt temperature	Melt, coolant and mold temperature

**Table 3:** Marked dimensions and dimensional tolerances of plastic parts.

Plastic part dimensioning (mm)		Plastic part size tolerance (mm) (tolerance size is 3 precision)
Dimensions	43.5	43.5_−0.33_^0^
	33.4	33.4_−0.29_^0^
	10.2	10.20_−0.17_^0^
	7	7_−0.15_^0^
	5.7	5.7_−0.13_^0^
	30.1	30.1_−0.29_^0^
	1.7	1.7_−0.11_^0^

Internal size	10	10_0_^+0.17^
	5	5_0_^+0.13^
	3.3	3.3_0_^+0.13^
	6.7	6.7_0_^+0.15^
	1.5	1.5_0_^+0.11^
	9.1	9.1_0_^+0.15^
	11.7	11.7_0_^+0.17^
	15.1	15.10_0_^+0.19^

**Table 4:** Radial and axial dimensions of cavity and core.

Plastic part size (mm)		Calculation formula	Core or cavity working size (mm)
Radial size of cavity	43.5_−0.34_^0^	A_M_+^*ξ*z^ = {(1 + S)A_S_-X△}^+*ξ*z^	43.46_0_^+0.11^
	33.4_−0.30_^0^		33.34_0_^+0.10^
	10.2_−0.18_^0^		10.12_0_^+0.06^
	5.7_−0.14_^0^		5.62_0_^+0.05^
	30.1_−0.30_^0^		30.03_0_^+0.10^
	1.7_−0.12_^0^		1.62_0_^+0.04^

Radial size of core	11.7_0_^+0.18^	B_M-*ξz*_ = {(1 + *s*)AS + *X*△}_-*ξz*_	11.89_−0.06_^0^
	5_0_^+0.14^		5.14_−0.05_^0^
	6.7_0_^+0.16^		5.85_−0.05_^0^
	1.5_0_^+0.12^		1.60_−0.04_^0^
	9.10_0_^+0.16^		9.27_−0.05_^0^

Radial size of cavity	7_−0.12_^0^	H_M_^+*ξ*z^ = {(1 + *s*)H_S_-2/3△}^+*ξz*^	6.96_−0.04_^0^

Axial dimension of cavity	2.4_0_^+0.14^	h_M-*ξz*_ = {(1 + *s*)h_S_+2/3△}^-*ξz*^	2.51_−0.05_^0^
	11.7_0_^+0.18^		11.88_−0.06_^0^
	15.1_0_^+0.20^		15.31_−0.07_^0^

**Table 5:** Maximum and minimum node displacement table.

Direction	Max displacement (mm)	Minimum displacement (mm)
*x*	0.003	−0.002
*y*	0.007	−0.005
*z*	0.007	−0.002

## Data Availability

The data set can be accessed upon request.
